# Estimation of return-to-sports-time for athletes with stress fracture – an approach combining risk level of fracture site with severity based on imaging

**DOI:** 10.1186/1471-2474-13-139

**Published:** 2012-08-06

**Authors:** Oliver Dobrindt, Birgit Hoffmeyer, Juri Ruf, Max Seidensticker, Ingo G Steffen, Frank Fischbach, Alina Zarva, Gero Wieners, Gerhard Ulrich, Christoph H Lohmann, Holger Amthauer

**Affiliations:** 1 Klinik für Radiologie und Nuklearmedizin, Universitätsklinikum Magdeburg A.ö.R. Otto-von-Guericke Universität, Leipziger Straße 44, Magdeburg 39120, Germany; 2 Orthopädische Universitätsklinik, Universitätsklinikum Magdeburg A.ö.R. Otto-von-Guericke Universität, Leipziger Straße 44, Magdeburg 39120, Germany

**Keywords:** Stress fracture, Grading system, Athletes, MRI, Bone scintigraphy

## Abstract

**Background:**

The aim was to compare the return-to-sports-time (RTST) following stress fractures on the basis of site and severity of injury. This retrospective study was set up at a single institution. Diagnosis was confirmed by an interdisciplinary adjudication panel and images were rated in a blinded-read setting.

**Methods:**

52 athletes (female, n = 30; male, n = 22; mean age, 22.8 years) with stress fracture (SFX) who had undergone at least one examination, either MRI or bone scintigraphy, were included. Magnetic resonance images (MRI) and/or bone scintigraphy (BS) of SFX were classified as either low- or high-grade SFX, according to existing grading systems. For MRI, high-grade SFX was defined as visibility of a fracture line or bone marrow edema in T1-, T2-weighted and short tau inversion recovery (STIR) sequences, with low-grade SFX showing no fracture line and bone marrow edema only in STIR and/or T2-weighted sequences. In BS images, a mild and poorly defined focal tracer uptake represented a low-grade lesion, whereas an intense and sharply marginated uptake marked a high-grade SFX. In addition, all injuries were categorized by location as high- or low-risk stress fractures. RTST was obtained from the clinical records. All patients were treated according to a non-weight-bearing treatment plan and comprehensive follow-up data was complete until full recovery. Two-sided Wilcoxon’s rank sum test was used for group comparisons.

**Results:**

High-risk SFX had a mean RTST of 132 days (d) [IQR 64d – 132d] compared to 119d [IQR 50d – 110d] for low-risk sites (p = 0.19). RTST was significantly longer (p = 0.01) in high-grade lesions [mean, 143d; IQR 66d – 134d] than in low-grade [mean, 95d; IQR 42d – 94d]. Analysis of high-risk SFX showed no difference in RTST (p = 0.45) between high- and low-grade [mean, 131d; IQR 72d – 123d vs. mean, 135d; IQR 63d – 132d]. In contrast, the difference was significant for low-risk SFX (p = 0.005) [low-grade; mean, 61d; IQR 35d – 78d vs. high-grade; mean, 153d; IQR 64d – 164d].

**Conclusion:**

For SFX at low-risk sites, the significant difference in RTST between low- and high-grade lesions allows more accurate estimation of RTST by this approach. Both location of the injury and severity determined by imaging should therefore be considered for prediction of RTST.

## Background

Due to the increasing number of recreational and elite athletes, sports related injuries pose an ongoing challenge for orthopedics and diagnosticians. Among these injuries, stress related fractures of the bone have gained increased recognition in recent years [1].

A stress fracture (SFX) is the result of repetitive overuse without adequate time for adaptation, which may lead to an accumulation of microfractures that exceed the remodeling-capacity of the bone [2]. Although the pathogenesis of a stress injury is multifactorial, the type of sport and stress applied has a major influence on their incidence and localization. The highest occurrences have been observed among long-distance and track athletes followed by gymnasts and field athletes [3,4]. Bones of the lower limb, especially the tibia, the metatarsals and the tarsal bones are affected most frequently [5]. The classification of stress fractures has been approached from two different directions. In orthopedics and sports medicine the fracture site is an important factor for clinical management. Stress injuries are classified as either high-risk or low-risk injuries, simply according to their location and the associated anatomic preconditions (e.g. increased tension in the osseous areas and constricted blood supply) [6,7].

The other approach to stress fracture classification is image-based grading of the severity of a lesion according to its appearance on magnetic resonance imaging (MRI) or bone scintigraphy (BS). Apart from mere diagnosis, image-based grading into high- and low-grade fractures can be used for the estimation of healing time and management of SFX [3,8,9].

The purpose of this study was to assess whether the combined analysis of the location of the injury and its severity, as determined by imaging, allows a more accurate prediction of return-to-sports-time (RTST) in stress fractures than estimations derived solely from image-based grading of fracture severity or risk level based on the site of injury.

## Methods

This retrospective analysis has been reviewed and approved by the ethics committee Otto-von-Guericke University Magdeburg with the assigned number 08/10.

### Patients and treatment

In this retrospective study 52 competitive athletes (male, n = 22; female, n = 30; mean age, 22.8 years (y), quartile(Q) (Q1, 16.0 y; Q2, 18.0 y; Q3, 24.8 y) with stress fractures were included (track, n = 18; long distance running, n = 16; handball, n = 13; soccer, n = 1; swimming n = 1; triathlon, n = 1; canoeing, n = 1; basketball, n = 1). All patients were either at a residential sports college or attending an Olympic training center or belonged to a professional sports team.

Treatment of all included patients took place at the Department of Sports Medicine, and was performed by the same team of doctors and physical therapists. Athletes presenting with symptoms suggestive of a stress injury were advised to immediately rest or minimize exercise of the affected site. Radiographs, MRI, and BS were used for initial diagnostic imaging. Only in cases of suspected aggravation or a delayed healing process, were follow-up examinations performed.

A confirmed stress injury was initially treated with an orthotic or a cast while the patient pursued a non-weight-bearing regime. The length of immobilization was dependent on the level of subjective and objective symptoms such as pain, swelling, restricted range of motion, and duration of symptoms prior to consultation. Of all 52 stress fractures, only two were treated surgically, one in the talar bone, the another in the navicular bone. Concomitantly, manual lymphatic drainage, two-cell baths and microcurrent therapy were used to reduce edema of the bone and the surrounding soft tissue. The main objective in the early stages of treatment was to prevent any pain. On that condition, alternative sports were allowed in order to maintain the level of fitness (e.g. swimming, biking, or monitored circle training in the gym) as well as proprioceptive training. In the later stages of treatment, sport-specific exercises were added to the training program. Athletes specialized in acceleration were helped to exercise explosive movements by physiotherapists, beginning with minimal weight bearing for very short periods. Athletes relying on quick and secure sideways movements practiced the likely motion processes of the respective discipline under a weight-reducing suspension. The weight and duration of the exercises were individually coordinated by the supervising doctors and physiotherapists. After progress in individual training, athletes were gradually reintegrated into their regular training program, starting with short sessions, which were prolonged according to the symptoms developing. Throughout the process, kinesiatrics, physical therapy, and occupational therapy were available to assist the athlete’s recovery.

### Imaging protocols

Magnetic resonance imaging (MRI) examinations were performed at 1.5 Tesla MRI (Intera 1.5 T MRI, Philips HealthCare, Best, The Netherlands). Depending on the location, axial, coronal and/or sagittal T1-weighted spin echo sequence (TR/TE: 400–600 ms/15–30 ms) as well as T2-weighted fast spin-echo sequence (TR/TE: 3,000 ms/44 ms, echo train 8) were acquired. In addition, a fat suppressed T2-weighted fast spin-echo sequence (TR/TE: 2,000–4,000 ms/60–80 ms, echo train 8) using short tau inversion recovery technique (STIR) or frequency selective chemical presaturation pulse was conducted. MRI examinations were performed with a field of view (FOV) of 160 × 160 mm – 240 × 240 mm, a matrix of 256 × 192 or 256 × 256, a slice thickness of between 3 mm and 5 mm, an interslice gap of between 0.4 mm and 3 mm, and with a number of excitations (NEX) of 1–2.

Three phases of bone scintigraphy images were obtained using a double head gamma-camera (e.cam, Siemens Healthcare, Erlangen, Germany) equipped with low-energy, high-resolution collimators. After the intravenous bolus-injection of 350–630 MBq (9.5-17.0 mCi) Technetium-99 m 3,3- diphosphono-1,2-propane dicarboxylic acid (Tc-99 m DPD) (Teceos; CIS bio international, GIF-sur-Yvette, France), planar data of the region of injury were recorded in a 64 × 64 matrix at a 1 second frame rate for the first minute. Static blood-pool images were obtained over the following 4 minutes. For the mineralization phase (3 h after injection), planar images were acquired over 5 minutes in the same view. Anterior and posterior scans were performed in all cases, a mediolateral for fibula and tibia and lateral images for feet were also acquired. In addition, anterior and posterior whole-body scans were performed at a table speed of 10 cm/min using a 256 × 1024 matrix.

### Data acquisition

For the purpose of this study, the following information was obtained on each patient from the records of the Department of Sports Medicine and the Department of Radiology and Nuclear Medicine: age, sex, type of sport, injury localization, beginning of symptoms, and the date of diagnostic imaging as bone scintigraphy (BS) and magnetic resonance imaging (MRI).

A reference standard was created by presenting each case to an adjudication panel consisting of experts in the fields of sports medicine, nuclear medicine, and radiology. All available data (clinical records and follow-up data, radionuclide imaging, MRI, and radiographs) were taken into consideration for the classification of a high- or low-risk injury and the determination of the exact time of return-to-sports. (Table 1) Return-to-sports was defined as the point in time, when the athlete was able to return to sports without restrictions and without any clinical or subjective symptoms.

**Table 1:** **High- and low-risk locations for SFX according to Boden et al. [**[6]**]**

**Anatomic region**	**High-risk locations**	**Low-risk locations**
Hip and femur	Femoral neck	Pelvis and femoral shaft
Knee and lower leg	Patella Anterior cortex tibia Medial malleolus tibiae	Proximal tibia Tibial shaft
Tarsal bones	Talus Tarsal navicular	Other tarsal bones
Mid- and forefoot	Fifth metatarsal Second metatarsal base Great toe sesamoid	Other metatarsal bones and digits

**Figure 1:**
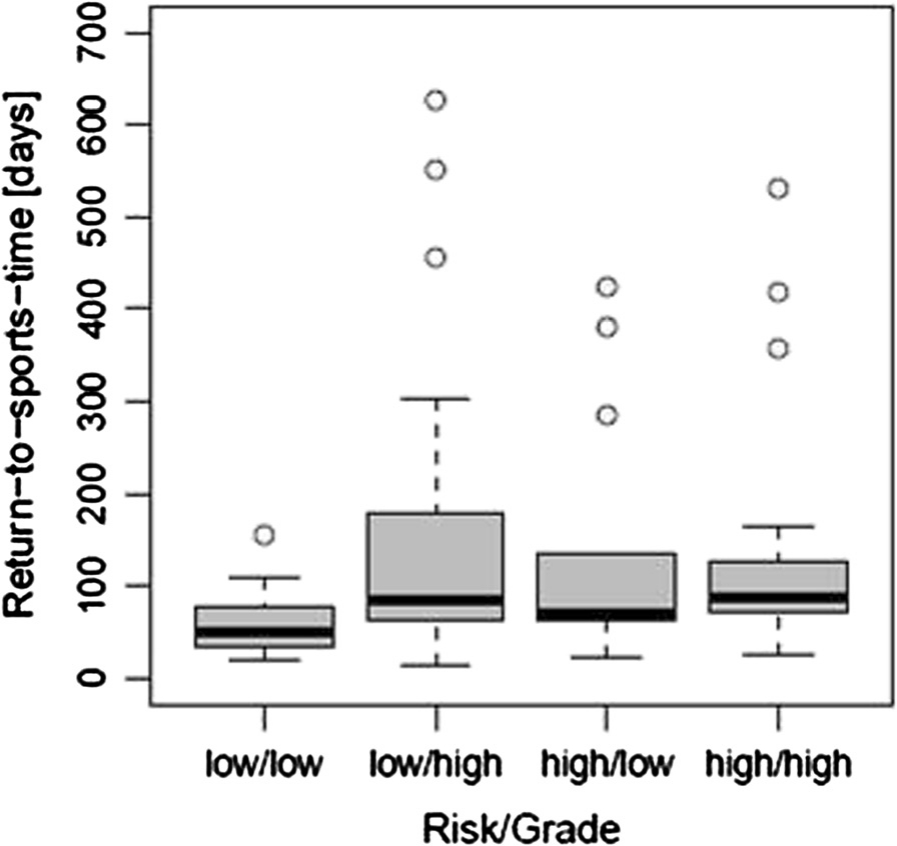
**Case of a high-risk, low-grade stress fracture Case of a 22-year-old male handball player with pain over the proximal fifth metatarsal bone. A**) shows bone marrow edema in T2-weighted MRI images in the transverse and coronal plane at the base of MT V. **B**) represents the corresponding T1-weighted images. The seemingly hypointense area indicated by the arrow was rated negative for bone marrow edema, showing no different signal intensity compared to the other metacarpal bases (not shown in displayed images). The anterior view of the osseous phase of bone scintigraphy **C**) shows a poorly defined area of increased uptake consistent with a low-grade injury. In accordance with our grading system (Table 1), this case was rated a low-grade stress injury at a high-risk site (Table 2) by the adjudication panel. The return-to-sports-time was recorded after 82 days.

Every patient underwent at least one examination with BS or MRI. BS images were rated in a separate blinded read setting by three independent specialists in nuclear medicine according to a simplified grading system for stress fractures derived from the work of Chisin et al. [8]. (Table 2). Similarly, MRI examinations were classified by three radiologists into low- and high-grade fractures following a simplified grading system proposed by Arendt et al. [5] (Table 2, Figure 1). Where a disagreement arose between the readers, a consensus was reached.

**Table 2:** Simplified grading systems for BS and MRI

	**Bone scintigraphy findings**	**MRI findings**
Low-grade stress fracture	**Irregular uptake **and/or a **poorly defined **area of increased activity, compared with the contralateral side	Bone marrow edema in STIR images, possibly in T2-weighted images
High-grade stress fracture	**Sharply marginated **area of increased activity, compared to the other side, usually focal or fusiform in shape	Bone marrow edema in T1- and T2-weighted image with or without a fracture line

Modified after Chisin et. al [8]. and Arendt et. al. [5].

### Statistical analysis

From every examination a dataset was created, containing the risk classification, the severity classification, and the return-to-sports-time measured from the date of the respective examination to the date of painfree return to full training. This included a total of 52 primary and 31 follow-up examinations at an interval of at least 6 weeks. Main outcome measurements were the estimation of return-to-sports-time depending on risk-classification (stress fracture at a low- or high risk site) and image-based grading (low- or high grade). Due to the small sample sizes a non-parametric distribution of the data was assumed and the healing times of all groups were compared using Wilcoxon’s rank sum test and Kuskal Wallis test. The reliability of the image-based grading was examined by calculating Fleiss' kappa.

R software, version 2.11.1 (The R Foundation for Statistical Computing, Vienna, Austria) was used for all statistical calculations.

All tests were two-sided and a P value lower than 0.05 was referred to as significant.

## Results

In this study 23/52 stress fractures were classified as a fracture at a high-risk site and 29/52 at a low-risk site (Tables 1 and 3). Dividing the examinations by injury location alone the corresponding return-to-sports-time (RTST) for high-risk fractures (n = 38), delivered a mean RTST of 132 days (d) [median 84 d, IQR (64 d - 132 d)] compared to 119 d [median 72 d, IQR (50 d - 110 d)] for low-risk site fractures (n = 45), (p = 0.19).

**Table 3:** Patient characteristics

	**Number (n)**	**Female/Male**	**Age (mean)**	**Type of sport (n)**	**Localization (n)**
Low-risk stress fractures	29	16/6	23.7	Distance running n = 12) Track (n = 9) Handball (n = 5) Other (n = 3)	Metatarsal (n = 14) Tarsal (n = 2) Tibia (n = 6) Fibula (n = 3) Other (n = 4)
High-risk stress fractures	23	14/16	21.7	Handball (n = 8) Track (n = 9) Long distance running (n = 4) Other (n = 2)	Metatarsal II (n = 3) Metatarsal V (n = 8) Navicular bone (n = 9) Talar bone (n = 2) Femur (n = 1)

Abbrevations: n, number.

Using image-based grading alone, RTST was significantly longer (p = 0.01) in stress fractures rated as high-grade lesions (n = 52) [mean 143 d, median 88 d, IQR (66 d - 134 d)] than in low-grade lesions (n = 31) [mean 95 d, median 64 d, IQR (42 d - 94 d)].

A combination of injury location and grading of severity based on imaging allows a further differentiation of stress fractures. By this two-dimensional approach, the examinations can be divided into four groups: 1) low-risk and low-grade (n = 17), 2) low-risk and high-grade (n = 28), 3) high-risk and low-grade (n = 14), 4) high-risk and high-grade (n = 24). The analysis of stress fractures only at high-risk sites showed no significant difference in RTST (p = 0.45) according to the imaging grading for high-grade lesions and low-grade lesions (mean, 135d vs. 131d).

**Figure 2:**
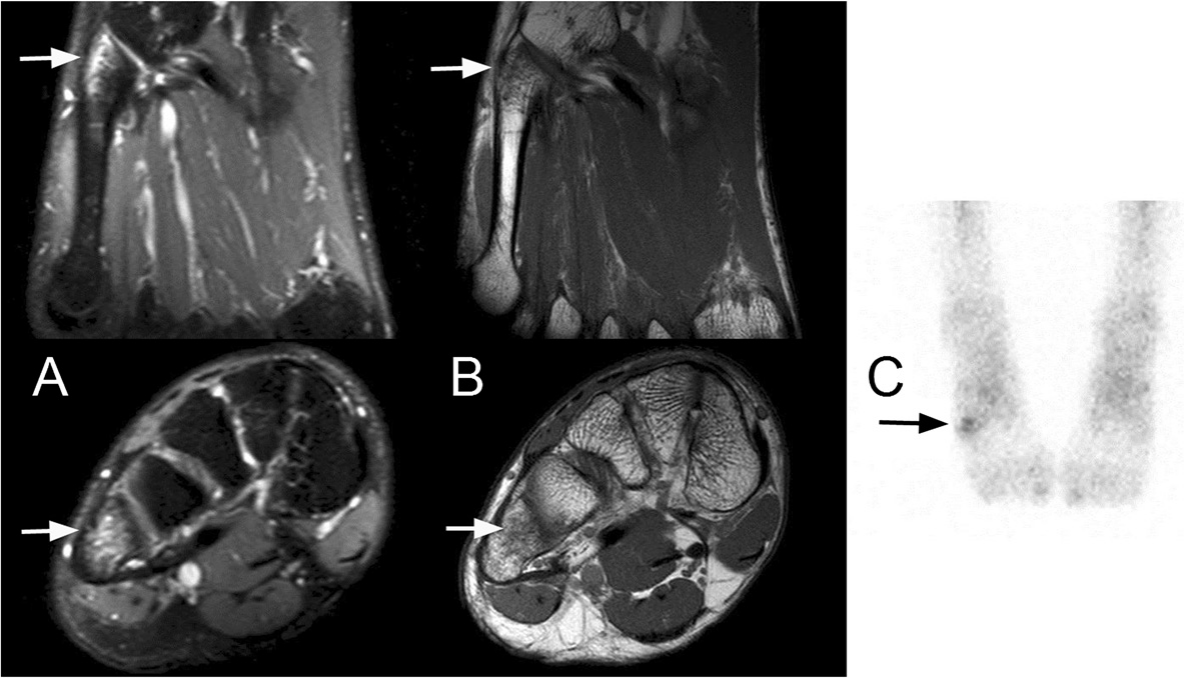
**Boxplots of RTST for stress fractures grouped according to risk and grade Boxplots of return-to-sports-time in days for groups according to site-based risk and image-based grading.** (low/low, low-risk/low-grade; low/high, low-risk/high-grade; high/low, high-risk/low-grade; high/high, high-risk/high-grade) Dots indicate outliers.

In contrast, the difference was significant for stress fractures at low-risk sites. Athletes with a SFX at low-risk sites and an imaging based low-grade lesion (n = 17), showed a mean RTST of 61 d [median 50 d, IQR (35 d - 78 d)] whereas for high-grade lesions (n = 28) at a high-risk location, a mean RTST of 153 d [median 86 d, IQR (64 d - 164 d)] was observed (p = 0.005). (Figure 2) (Table 4) Furthermore, the RTST of the group with low-grade and low-risk fractures differed significantly to that of all the other three groups. (Table 5) Between these three other groups no significant difference in RTST could be observed. The interobserver reliability of the readers, classifying the images as either high- or low-grade fractures was k = 0.83 for BS and k = 0.82 for MRI, respectively.

**Table 4:** Statistical distribution of RTST for SFX grouped according to severity and risk level of location

	**Mean (days)**	**Median (days)**	**Q 25 (days)**	**Q 75 (days)**
LowRiskLowGrade	61	50	35	78
LowRiskHighGrade	153	86	64	164
HighRiskLowGrade	135	70	63	132
HighRiskHighGrade	131	89	72	124

Abbreviation: Q, quartile.

**Table 5:** Statistical comparison of low-risk/low-grade SFX to all other groups

	**low-high**	**high-low**	**high-high**
low-low	0.005	0.02	0.01

(low/low, low-risk/low-grade; low/high, low-risk/high-grade; high/low, high-risk/low-grade; high/high, high-risk/high-grade).

## Discussion

This study demonstrated the ability of functional imaging to predict the return-to-sports-time of athletes after a stress fracture. For this purpose, both the severity and location of the injury need to be determined. It could be shown that patients with stress fractures of low-grade and low-risk required significantly shorter RTSTs than patients with high-grade and/or high-risk stress fractures.

An estimation of return-to-sports-time plays an important role in the management and treatment of high performance athletes [6,10,11]. Therefore a simple and reliable method of estimating the healing time of stress fractures is desirable. Due to the lack of sensitivity of plain radiographs, accurate diagnosis relies heavily on either magnetic resonance imaging (MRI) or bone scintigraphy (BS) [1]. Both methods have been found to deliver reliable results of equivalent accuracy, with BS appearing to provide a slightly higher sensitivity, and MRI a higher specificity for the diagnosis of a stress fracture [12-15].

In our study, stress fractures were rated as either high- or low-grade, using MRI and BS as equally suitable imaging modalities for the classification. Despite their use of different imaging modalities, Ishibashi et. al [16]. showed an agreement of 86.1% in a prospective study of 36 cases, while Fredericson et. al [17]. showed an agreement of 77.8% between MRI-grading and BS-grading in stress fractures. Both studies used a 4-point scale, from low- to high-grades of fracture. This study used a simplified 2-grade grading-system for BS and MRI, combining grade 1 and 2 to low-grade stress fractures and combining grade 3 and 4 to high-grade stress fractures. A previous study of ours demonstrated the usefulness of a simplification of the BS grading-system, showing a significant difference between high- and low-grade SFX. Furthermore, a close correlation was demonstrated between the healing times for grade 1 and 2 fractures as well as for the grades 3 and 4 [12]. Arendt et al. have also shown a significant difference in healing time between high-grade and low-grade stress injuries using their MRI grading-system [5]. This approach provides easier grading, satisfactory accuracy, and results of statistical significance. The almost perfect interobserver agreement (>0.80) for the grading of stress fractures by both modalities emphasizes the robustness and reliability of the chosen, simplified grading systems [18].

With regard to the correlation of RTST and image-based grading, published results are contradictory. Studies failing to show a significant correlation have been published by Dutton et. al [19] retrospectively evaluating 37 BS images of tibiae with stress fractures, Yao. et. al [20] examining 35 stress fractures using an MRI grading system, and Fredericson et. al. investigating an MRI grading system for stress fractures with a study of 18 symptomatic tibiae [17].

In contrast, a significant difference in RTST between high- and low-grade stress fractures was shown by Arendt et. al. who retrospectively compared the healing time of athletes (n = 61) recovering from a stress fracture with an MRI-grading system [5]. In a previous analysis evaluating a BS grading-system, our group was able to demonstrate a significant difference in healing time between high- and low-grade lesions [12].

One reason for these discrepant results might be that all the aforementioned studies graded stress fractures on imaging alone, independent of the site of injury. For example, the tibia, a frequent and well-examined stress fracture site, contains multiple high-risk sites (anterior midshaft or medial malleolus = high-risk, posteromedial aspect = low-risk) [5,6,19,20]. Recent case reports and publications therefore emphasize the need to differentiate between high- and low-risk sites for treatment and estimation of RTST [10,11,21].

To our knowledge, the present analysis is the first study to assess RTST and grading based on MRI or bone scintigraphy in combination with SFX risk-classification.

According to our data, only stress fractures classified as low-grade and low-risk have a healing time that is significantly shorter than all other measured categories. As soon as a stress fracture is either high-risk or high-grade, it can be assumed that the course of recovery will be prolonged.

It is reported that higher rates of nonunion and complications delay the healing time of injuries at high-risk sites [10,11]. Reviews in the literature also state that complications involving prolonged healing are rare in the group of low-risk fractures [10,11]. Though no complications or specific reasons could be identified with certainty in every case of this retrospective study, prolonged healing times of more than 200 days were seen in all categories but the low-risk/low-grade group. (Figure 2) This again underlines the different character of the low-risk/low-grade group.

These findings show that early diagnosis is of paramount importance and progression from a low-grade SFX to a high-grade SFX has to be avoided by early intervention. For high-risk injuries a more aggressive treatment with stringent restrictions is advisable, regardless of the severity. Immobilization should possibly be prolonged, despite the absence of symptoms, but to answer this question adequately, prospective studies first need to be performed. The group of low-risk and low-grade stress fractures did not show complications under the presented treatment-plan, but in order to avoid progression of the injury, an accelerated reintegration into training cannot generally be recommended. In a previous study, a proportion of patients with low-grade stress fractures were found to recover under ongoing stress, while in others the injury progressed to a high-grade fracture [8]. Therefore the decision has to be made individually and in consultation with the athlete.

Follow-up examinations may be useful to monitor the healing process, in which case we propose MRI, as it does not involve exposure to radiation. In the case of an inconclusive initial MRI, or a negative follow-up MRI with persisting clinical symptoms, we propose bone scintigraphy, due to its higher sensitivity and high negative predictive value.

Certain limitations arising from the retrospective setting of this study should be mentioned. Treatment was not entirely standardized and clinical suspicion influenced some decisions regarding time of immobilization, but with all the patients being competitive athletes, the aim was nevertheless, to achieve the quickest possible return to sports. Furthermore, the cases of stress fractures presented form a heterogeneous group with regard to location, which may limit the quality of the conclusion. On the other hand, a general statement on the RTST of high- and low-risk SFX can only be drawn from a study assessing a large variety of different locations. Finally, the return-to-sports-time was determined clinically and not confirmed with imaging follow-up examinations, which would be desirable.

Still, we believe that these results make a useful contribution to current sports medicine practice. To our knowledge, no prospective study evaluating RTST of stress fractures has yet been published, though such a study would be of great interest.

## Conclusion

Stress fractures show a prolonged healing-time at high-risk sites, irrespective of the severity grade based on imaging, and at low-risk sites displaying a high-grade lesion on imaging. As a consequence, both the risk level of the fracture site and the grade of a stress fracture based on imaging should be considered for prediction of RTST. These findings underline the importance of fast and reliable diagnosis to prevent possible progression of a low-grade stress injury to a high-grade fracture. Moreover, the necessary information for this more differentiated approach to estimating RTST can be reliably obtained within a single examination. This straightforward classification is useful for clinical practice, since it is easy to apply and memorize.

## Abbreviations

RTST: Return-To-Sports-Time; SFX: Stress Fracture; BS: Bone Scintigraphy/Bone Scan; MRI: Magnetic Resonance Imaging; y: years; Q: Quartile; IQR: Inter Quartile Range; STIR: Short Tau Inversion Recovery; k: kappa; p: probability.

## Competing interests

The authors declare that they have no competing interests.

## Authors’ contributions

OD: study concept, data acquisition, data analysis, manuscript writing, final approval. BH: study concept, member of adjudication panel, manuscript revising, final approval. JR: blinded reader, manuscript revising, final approval. MS: blinded reader, manuscript revising, final approval. IS: statistical analysis, data analysis, manuscript revising, final approval. FF: blinded reader, member of adjudication panel, data analysis, manuscript revising, final approval. AZ: blinded reader, manuscript revising, final approval. GW: blinded reader, manuscript revising, final approval. GU: blinded reader, manuscript revising, final approval. CL: member of adjudication panel, data analysis, manuscript revising, final approval. HA: study concept, member of adjudication panel, data analysis, manuscript writing and revising, final approval.

## Pre-publication history

The pre-publication history for this paper can be accessed here:

http://www.biomedcentral.com/1471-2474/13/139/prepub
